# Perceived barriers to the implementation of social distancing in the COVID‐19 pandemic in Iran during 2020–2021: A cross‐sectional study

**DOI:** 10.1002/hsr2.947

**Published:** 2022-11-21

**Authors:** Abdolreza Gilavand, Craig S. Webster

**Affiliations:** ^1^ Social Determinants of Health Research Center Ahvaz Jundishapur University of Medical Sciences Ahvaz Iran; ^2^ Department of Education Development Center Ahvaz Jundishapur University of Medical Sciences Ahvaz Iran; ^3^ Department of Anaesthesiology and Center for Medical and Health Sciences Education, School of Medicine University of Auckland Auckland New Zealand

**Keywords:** barriers, COVID‐19, Iran, pandemic, social distancing

## Abstract

**Background and Aims:**

This research has been on the effective role of social distancing in preventing the spread of COVID‐19 and the obstacles to its implementation. The results of this research can highlight the major barriers to distancing and suggest appropriate solutions to remove them.

**Methods:**

We conducted this cross‐sectional study during 2020–2021 among 277 faculty members, students of medical universities and ordinary people of Khuzestan province in southwestern Iran. We included them in this study by sampling at convenience. The data collection tool was a researcher‐constructed questionnaire that we distributed among the statistical sample through social networks (WhatsApp and Telegram).

**Results:**

Mean ± SD = economic barriers 4.49 (0.65), cultural barriers 4.48 (0.70), social barriers 4.40 (0.61), political barriers 4.28 (0.64), educational barriers in universities and schools 4.27 (0.53) and Educational barriers at societal level 3.82 (1.08) were the self‐reported obstacles (perceived) to social distancing during the Covid‐19 pandemic. Comparison of occupational groups with faculty members showed that only scores of academic barriers have a significant difference between occupational groups (*p* < 0.001).

**Conclusion:**

The role of economic barriers, cultural barriers and social barriers in social distancing was very prominent. One of our remarkable results was that there is adequate training for people on the proper implementation of the principles of social distance in the prevention of the Covid‐19 pandemic. The responsibility of all members of society to observe social distancing as a moral and even legal duty can be the first step to protect the health of citizens against COVID‐19. We can, therefore, use some planned interventions. This must be within the framework of economic, cultural, social and political structures of society.

## 1 INTRODUCTION

The coronavirus was reported in mid‐December 2019 in Wuhan, central China, and on March 11, 2020, the World Health Organization declared the outbreak a pandemic.^1^, ^2^, ^3^, ^4^, ^5^, ^6^, ^7^, ^8^ Since then, the Covid‐19 virus has infected millions of people worldwide and killed millions.^1^ The emergence of the Covid‐19 pandemic and its outcomes have led to fear, concern and anxiety among people around the world.^8^ Studies have shown that nonpharmacological measures (physical distance, face mask and eye protection to prevent transmission of the virus) can be very effective in reducing the spread of coronavirus. Observing a distance of at least 1 meter clearly reduces the risk of infection, and the greater the distancing between people (2 m for example), the more will be its effectiveness.^1^ Studies have shown that although social distancing is supposedly one of the most effective nonpharmacological measures to reduce the transmission of Covid‐19 pandemic, doing so may face many challenges. Unfortunately, these difficulties are likely to reduce adherence to social distancing measures and thereby reduce their effectiveness^2^, ^3^ In addition, economic considerations as well as concerns about the development of mental disorders due to social distancing have prevented its observance.^9^ Covid‐19 has created some changes in people's lifestyles. The most common of these are avoiding social gatherings, obsessive washing of hands and storing essential food and supplies.^9^

The National Headquarters for Management and Control of Corona in Iran, as in other parts of the world, has launched a social distancing plan to manage the spread of this virus. Social distancing means creating a physical distance between individuals and preventing social gatherings to maintain individual and public health. As recommended, the use of this strategy should not lead to the destruction of social interactions and it means only physical distance. Perhaps it is better to use the word physical distancing instead of social distancing in order for people to communicate with each other. Extensive researches around the world have shown that the application of social distancing and home quarantine is the most important working procedure to prevent the spread of the Covid‐19 pandemic. Considering the specific cultural, social, economic, political structure of Iranian society, recognizing major barriers to social distancing is critical to improving the prevention of the spread of this deadly virus. Our research investigates the self‐reported barriers to the social distancing requirements in response to the Covid‐19 pandemic in Khuzestan province in southwestern Iran. The results of this research can highlight the major barriers to distancing and suggest appropriate solutions to remove them.

## 2 MATERIALS AND METHODS

This cross‐sectional research (2021) investigates the self‐reported obstacles to the social distancing in the Covid‐19 pandemic in Khuzestan province in southwestern Iran. The population of this research consisted of faculty members, students of medical universities and ordinary people of Khuzestan province. In this research, Sampling was done with convenience sampling, recruiting 277 people. The faculty members of medical sciences universities were considered as the reference group. However, from the opinions of 33 students and 104 employees in universities of medical sciences, as well as 47 other people who either did not have a job or were not working in government offices, they were identified as experts and their opinions were used. Data collection tool is researcher‐constructed (in Persian) and a multidimensional questionnaire (38 questions) including demographic characteristics and major barriers to social distancing in pandemic Covid‐19. These barriers assess the major barriers to social distancing of Pandemic Covid‐19. They are cultural factors (8 questions), economic factors (4 questions), political factors (9 questions) Social factors (7 questions), educational factors at the society (question 3) and academic factors in universities and schools (6 questions). Their scoring is on a 5‐grade Likert scale (where 1 = *I completely disagree*, 2 = *I disagree*, 3 = *I have no opinion*, 4 = *I agree*, and 5 = *I completely agree*). In this research, we examined and compared the barriers of social distancing separately according to the relevant field and considering the statistical sample. The validity and reliability of this questionnaire has received confirmation. The experts have confirmed its content validity. We provided the questionnaire to five clinical and health specialists of the university and the panel of experts approved both the index value and content validity rate for all questions. All five experts approved the questions in terms of necessity, relevance, simplicity and clarity. We used re‐testing to determine the reliability of the instrument. We distributed 20 questionnaires with a time interval of 2 weeks among faculty members, students and ordinary people and examined the reliability of the questions. With the help of Pearson correlation test, we obtained a significant relationship by 86%. The reliability of the structure was confirmed by Cronbach's alpha at 92% agreement. This questionnaire was structurally evaluated by exploratory factor analysis and after going through validity steps with CVI and CVR indices equal to 89% and 87%, respectively. We distributed this questionnaire through statistical networks among the statistical sample. The designed electronic questionnaire was sent through specialized and general WhatsApp and Telegram groups that could be accessed directly or indirectly. To comply with ethical considerations and before sending the questionnaires, we explained the objectives of the study and the sensitivity of receiving accurate answers to the statistical sample. After obtaining their consent, we assured them that researchers consider the received information as confidential. Inclusion criteria were being a faculty member, student or ordinary citizen of Khuzestan province who were willing to participate in the study. Exclusion criterion was cancellation of participation in the study. We performed Data analysis through SPSS (SPSS 24, Inc.) at a significance level of 0.05. We used mean and standard deviation to describe the observations, frequency for continuous variables and percentage for class variables. Kolmogorov–Smirnov test confirmed the normality of the distribution of observations. To evaluate the mean scores of the questionnaire in the levels of binary variables, we used independent t‐test and analysis of variance and Dunnett's test for variables with levels of more than 2 classes. This study was funded by Ahvaz Jundishapur University of Medical Sciences with number SDH‐9930 and ethics code IR.AJUMS.REC.1399.617.

## 3 RESULTS

Table 1 shows the personal characteristics of the participants, from which we can see that 277 people participated in this research. Of which 93 were faculty members, 33 were students, and 104 were employees in universities of medical sciences. Also, 47 of them were either unemployed or not working in government offices and were identified as experts and their opinions were used in this research. Also, 43.8% of the participants were women and 56.2% were men. A total of 24.1% were single and 75.9% were married. In terms of age, 0.7% of them were under 20 years old, 17.8% between 21 and 30, 34.5% between 31 and 40, 35.3% between 41 and 50, 10.2% between 51 and 60 and 1.5% over 60 years old.

**Table 1:** Personal characteristics of the participants^a^

Questions	Frequency	Percentage
Gender		
Female	121	43.8
Male	155	56.2
Marital status		
Single	63	24.1
Married	198	75.9
Education		
Under diploma	0	0.0
Diploma	24	8.6
Associate degree	21	7.5
Bachelor	63	22.6
Master	53	19.0
PhD or specialty	107	38.4
Sub‐specialty	11	3.9
Age		
<20	2	0.7
21–30	49	17.8
31–40	95	34.5
41–50	97	35.3
51–60	28	10.2
>60	4	1.5
Occupation		
Faculty members	93	33.6
Government's employee	104	37.5
Nongovernment job	25	9.0
Unemployed	22	7.9
Student	33	11.9

a Categories which fail to add to 277 indicate questions that participants declined to answer.

According to Table 2 and taking into account the occupational status of the participants, we identified the following factors as the main barriers to social distancing during the Covid‐19 pandemic, respectively. Mean ± SD = economic barriers 4.49 (0.65), cultural barriers 4.48 (0.70), social barriers 4.40 (0.61), political barriers 4.28 (0.64), educational barriers in universities and schools 4.27 (0.53) and Educational barriers at the societal level 3.82 (1.08) were the self‐reported obstacles (perceived) to social distancing during the Covid‐19 pandemic. Educational barriers at the societal level were at the lowest level. The faculty members also mentioned cultural barriers 4.58 (0.62) and economic barriers 4.52 (0.66) as the main barriers to social distancing, respectively. Unemployed people mentioned social factors 4.52 (0.48) and political factors 4.45 (0.50), respectively. Students mentioned educational barriers 4.08 (0.96) and those who were nongovernmental jobs mentioned academic barriers 4.21 (0.73).

**Table 2:** Obstacles to distancing according to the occupational status of the participants

	Frequency	Mean	Standard deviation	Standard error
Cultural				
Faculty members	93	4.5745	0.61839	0.06412
Government's employee	104	4.4179	0.80583	0.07902
Nongovernment job	25	4.5173	0.52942	0.10588
Unemployed	22	4.4765	0.65434	0.13951
Student	33	4.3808	0.70322	0.12241
All observations	277	4.4797	0.69990	0.04205
Economic				
Faculty members	93	4.5215	0.66518	0.06898
Government's employee	104	4.4832	0.70990	0.06961
Nongovernment job	25	4.5200	0.51498	0.10300
Unemployed	22	4.5227	0.53402	0.11385
Student	33	4.3561	0.60606	0.10550
All observations	277	4.4874	0.65235	0.03920
Political				
Faculty members	93	4.2266	0.63531	0.06588
Government's employee	104	4.3237	0.69053	0.06771
Nongovernment job	25	4.2133	0.62189	0.12438
Unemployed	22	4.4520	0.50170	0.10696
Student	33	4.2391	0.63411	0.11038
All observations	277	4.2812	0.64539	0.03878
Social				
Faculty members	93	4.4215	0.59378	0.06157
Government's employee	104	4.4308	0.63231	0.06200
Nongovernment job	25	4.1600	0.74386	0.14877
Unemployed	22	4.5273	0.48026	0.10239
Student	33	4.3030	0.52469	0.09134
All observations	277	4.3957	0.61028	0.03667
Educational				
Faculty members	93	3.6774	0.98534	0.10218
Government's employee	104	3.8029	1.19143	0.11683
Nongovernment job	25	4.0600	0.89350	0.17870
Unemployed	22	3.8182	1.17053	0.24956
Student	33	4.0758	0.96113	0.16731
All observations	277	3.8177	1.07431	0.06455
Academic				
Faculty members	93	3.5712	0.90052	0.09338
Government's employee	104	4.0747	0.87171	0.08548
Nongovernment job	25	4.2150	0.73467	0.14693
Unemployed	22	4.0114	1.01617	0.21665
Student	33	4.1061	0.61167	0.10648
All observations	277	3.9170	0.88626	0.05325
Questionnaire				
Faculty members	93	4.2239	0.47686	0.04945
Government's employee	104	4.2787	0.57200	0.05609
Nongovernment job	25	4.3147	0.48524	0.09705
Unemployed	22	4.3264	0.55012	0.11728
Student	33	4.2631	0.45549	0.07929
All observations	277	4.2655	0.51635	0.03102

Table 3 compared other occupational groups with faculty members of medical universities that only scores of academic barriers showed a significant difference between occupational groups.

**Table 3:** Comparison of occupational groups

	One‐way analysis of variance				
	Total squares	Degree of freedom	Mean total squares	Fisher statistic	*p*
Cultural					
Between classes	1.591	4	0.398	0.810	0.520
Within classes	133.609	272	0.491		
All observations	135.201	276			
Economic					
Between classes	0.733	4	0.183	0.427	0.789
Within classes	116.722	272	0.429		
All observations	117.456	276			
Political					
Between classes	1.281	4	0.320	0.767	0.548
Within classes	113.681	272	0.418		
All observations	114.963	276			
Social					
Between classes	2.243	4	0.561	1.517	0.197
Within classes	100.552	272	0.370		
All observations	102.795	276			
Educational					
Between classes	5.518	4	1.380	1.199	0.312
Within classes	313.025	272	1.151		
All observations	318.543	276			
Academic					
Between classes	17.300	4	4.325	5.897	0.000
Within classes	199.485	272	0.733		
All observations	216.786	276			
Questionnaire					
Between classes	0.322	4	0.080	0.298	0.879
Within classes	73.266	272	0.269		
All observations	73.587	276			

Dunnett's test showed that the scores of the academic barriers of the faculty members were significantly lower than the scores of government employees, students, and nongovernmental jobs (Table 4).

**Table 4:** Significance of occupational groups

	Occupation (I)	Occupation (J)	Mean difference (I–J)	Standard error	*p*
Academic obstacles	Government's employee	Faculty members	0.50345	0.12222	0.000
	Nongovernment job	Faculty members	0.64376	0.19293	0.004
	Unemployed	Faculty members	0.44013	0.20303	0.112
	Student	Faculty members	0.53482	0.17352	0.009

The results of the multiple linear regression model, taking into account the adjusted coefficient of the variables on the educational barriers score of the samples, showed that gender, age and marital status had no effect on their score. However, considering the educational category below the diploma as the Reference group of education variable, it was seen that the average academic score of people with bachelor's and postgraduate education is 0.535 on average (standard error = 0.233, significance = 0.022) and 638 0/0 (standard error = 0.273, significance = 0.020) is more than people with less than diploma education. Also, faculty members had a significantly lower average score of educational factors barriers than students, Nongovernment job and government's employee. The results are presented in Table 5.

**Table 5:** The results of the multiple linear regression model considering the variable of academic score as the response variable and the variables of age, gender, marital status, and education as auxiliary variables

Variables	Coefficient of determination	Standard Error	Coefficient wald statistic	*p* value
y‐Intercept	3.393	0.6998	23.514	0.000
Gender (male)	0.136	0.1138	1.432	0.231
Marital status (married)	−0.013	0.1394	0.009	0.924
Education				
Sub‐specialty	0.603	0.3760	2.575	0.109
PhD or specialty	0.494	0.2627	3.536	0.060
Bachelor	0.535	0.2332	5.264	0.022
Master	0.426	0.2342	3.313	0.069
Associate degree	0.636	0.2739	5.389	0.020
Under diploma	Reference group			
Age				
>60	0.032	0.7945	0.002	0.968
51–60	−0.307	0.6493	0.224	0.636
41–50	−0.468	0.6338	0.546	0.460
31–40	−0.399	0.6340	0.397	0.529
21–30	−0.373	0.6408	0.340	0.560
<20	Reference group			
Occupation				
Student	0.574	0.2419	5.627	0.018
Unemployed	0.532	0.2805	3.600	0.058
Nongovernment job	0.837	0.2659	9.903	0.002
Government's employee	0.526	0.1831	8.237	0.004
Faculty members	Reference group			

Pearson correlation test showed that there is a significant and direct relationship between the inhibiting factors. The results are presented in Table 6.

**Table 6:** The correlation between the barriers factors

		Economic	Political	Social	Educational	Academic	Cultural
Economic		1	0.495	0.250	0.219	0.232	0.414
	*p*		0.000	0.000	0.000	0.000	0.000
Political			1	0.397	0.424	0.481	0.333
	*p*			0.000	0.000	0.000	0.000
Social				1	0.313	0.280	0.316
					0.000	0.000	0.000
Educational					1	0.630	0.104
						0.000	0.082
Academic						1	0.154
							0.010
Cultural							1

## 4 DISCUSSION

Respondents gave high scores on all dimensions of the questionnaire. The main obstacles to social distancing in the Covid‐19 Pandemic in Iran were economic barriers, cultural, social, political barriers, academic obstacles in the universities and schools, and educational obstacles at societal level. Therefore, we can claim that this research was able to examine the major obstacles. In their research in Brazil, Thomé et al.^6^ divided the barriers to social distancing into three political, socioeconomic, and scientific dimensions, the results of which are consistent with our research. Coroiu et al.^10^ conducted a study among English‐speaking adults in Europe and North America. They have cited the social barriers (excessive walking in the streets, communication with family and friends and the stress of isolation and loneliness), political barriers (lack of trust in government messages about the epidemic) as the main obstacles to social distancing. In this research, in terms of social distance, men and young people showed less commitment than the women and the elderly. In their research in Ethiopia, Hailu et al.^11^ cites political barriers (poor compliance with social distancing measures by the government and health authorities to prevent COVID‐19) as the most important reasons for noncompliance with social distancing. The observance of social distancing increased with age. In a systematic review article, Sadjadi et al.^12^ examined the major barriers to implementing social distance. In this study, they examined 29 articles and mentioned the psychosocial phenomena at the individual or social level and shortcomings in government action or communication as barriers to social distancing. In a research conducted in Ireland, Berry et al.^13^ cited the lack of environmental support for social distancing, the observation of other people who do not observe social distancing, and the lack of physical interaction by others as the most important causes of failure in social distancing, respectively. Most of the participants in this research were young and students. In their research in Spain, for Gonzalez et al.^14^ have cited other factors. The “fear of the effects of distancing on general behavior (such as mental health), feelings of isolation and depression due to distance from family and friends, physical health threats due to conditioning and food insecurity, financial problems that have caused sudden and permanent unemployment and imposed unexpected costs on individuals during the Covid‐19 pandemic” are barriers to social distancing.

Among other significant results of this research, it can be mentioned that educational barriers at the societal level were at the lowest level, which indicates that there is training for people on the proper implementation of the principles of social distance in the prevention of the Covid‐19 pandemic. Also, the scores of the academic barriers of the faculty members were significantly lower than the scores of government employees, students and nongovernmental jobs. which shows that some measures such as virtual education, smart social distancing in classes, conference halls and public spaces of universities have been able to reduce unusual gatherings during the Covid‐19 pandemic in universities.

Although the practice of social distancing has been a key measure to reduce COVID‐19 transmission, the implementation of strict measures for social distancing is very challenging. Given its urbanization level and social and religious norms and its annual hosting of large international religious communities, Saudi Arabia has begun significant measures of social distancing. These mainly include the suspension or cancellation of religious gatherings, entertainment and sports, and events such as the Hajj, the temporary closure of educational centers and mosques, and the postponement of all unnecessary assemblies. It has been in favor of the interest of public and global health despite its social, economic, political and religious challenges.^3^ Iran, too, has taken such measures due to some social, religious and cultural similarities. According to Uscher et al.,^4^ applying social distancing in schools and educational settings will face many challenges. Thomé et al. examined the benefits of social distance in slowing the spread of COVID‐19 in the United States and showed that although social distance saves lives, it imposes costs on society due to reduced economic activity.^6^ Social distancing puts pressure on the basic human need to communicate with each other. The recommendation of health organizations to limit close human communication may cause mental health problems after the epidemic and even during it such as depression, anxiety and domestic violence. As arms and ammunition sales increase in the United States, so in Switzerland, the judiciary is preparing itself for increasing domestic violence. The provision of health services will be even more challenging.^2^

For proper observance of social distancing in society, people's adherence to restrictive measures, general acceptance of health advice and financial level of countries are necessary.^6^ Oosterhoff et al.^5^ conducted a research in the United States with 683 participants in the research through social media. The results showed that 98.1% of them are at the lowest level of social distancing. Social responsibility and avoiding the desire to get sick were the main motivations for observing social distance. In a research, creating opportunities and foundations for social distancing, increasing awareness and motivation against the costs of nondistancing and increasing opportunities to see others adhere to the guidelines were for Berry et al.^13^ the most important facilitators for improvement of social distancing.

Research of Block et al.^7^ at the University of Oxford showed that the best way to reduce the spread of the coronavirus was to limit daily interactions to more or less repetitive contact with a small group. These limited domains of communication are termed “social bubbles.” This strategy, based on reducing day‐to‐day contacts, can significantly reduce the incidence of Covid‐19 and lower its growth curve beyond simple social distancing. This means that people are only temporarily and in times of crisis in contact with people who have similar characteristics or living space, such as people living in the same neighborhood. On the other hand, the results of this research have determined that to reduce the extent of Covid‐19 outbreaks, it is better to reduce and limit the interaction with distant acquaintances that we see occasionally. In other words, reducing strategically the scope of contacts also increases the effectiveness of other measures like social distancing. Thus, in addition to social distance conditions, it is possible to make some contacts with less risk. This approach can also reduce the negative consequences of social isolation. They also believe that strategic and selective social distancing, or social bubbles, while reducing the extent of the epidemic, can also reduce the psychological and economic damage of long‐term quarantine. People are also more likely to comply with such restrictions, as it is more satisfactory than strict quarantines and complete isolation. These days, many countries that have been the epicenter of the corona outbreak are gradually dismantling quarantine regulations, but their authorities continue to emphasize the need for social distancing.^7^

## 5 CONCLUSION

The role of economic barriers, cultural barriers and social barriers in social distancing was very prominent in our results. Humans are social beings. Regardless of nationality or cultural background, maintaining isolation for a long time can cause considerable psychological distress. The economic burden of this epidemic with millions of lost jobs, rising poverty and inequality may exacerbate these feelings. This status may be greater in developing countries. One of the remarkable of this research was that there is adequate training for people on the proper implementation of the principles of social distance in the prevention of the Covid‐19 pandemic. The responsibility of all members of society to observe social distance as a moral and even legal duty can be the first step to protect the health of citizens against COVID‐19. We can use some planned and successful interventions. This must be within the framework of economic, cultural, social and political structures of society.

## AUTHOR CONTRIBUTIONS

**Abdolreza Gilavand**: conceptualization; data curation; formal analysis; funding acquisition; investigation; methodology; project administration; resources; software; supervision; validation; visualization; writing – original draft; writing – review & editing. **Craig S Webster**: writing – review & editing. All authors have read and approved the final version of the manuscript. The corresponding author had full access to all of the data in this study and takes complete responsibility for the integrity of the data and the accuracy of the data analysis.

## CONFLICTS OF INTEREST

The authors declare no conflicts of interest.

## TRANSPARENCY STATEMENT

The lead author Abdolreza Gilavand affirms that this manuscript is an honest, accurate, and transparent account of the study being reported; that no important aspects of the study have been omitted; and that any discrepancies from the study as planned (and, if relevant, registered) have been explained.

## Data Availability

The original contributions presented in the study are included in the article/supplementary material, further inquiries can be directed to the corresponding author.
